# Proximity to agriculture is correlated with pesticide tolerance: evidence for the evolution of amphibian resistance to modern pesticides

**DOI:** 10.1111/eva.12069

**Published:** 2013-04-30

**Authors:** Rickey D. Cothran, Jenise M. Brown, Rick A. Relyea

**Affiliations:** ^1^ Department of Biological Sciences and Pymatuning Laboratory of Ecology University of Pittsburgh Pittsburgh PA USA; ^2^ Present address: Department of Integrative Biology University of South Florida Tampa FL 33620 USA

**Keywords:** adaptation, ecotoxicology, life history evolution

## Abstract

Anthropogenic environmental change is a powerful and ubiquitous evolutionary force, so it is critical that we determine the extent to which organisms can evolve in response to anthropogenic environmental change and whether these evolutionary responses have associated costs. This issue is particularly relevant for species of conservation concern including many amphibians, which are experiencing global declines from many causes including widespread exposure to agrochemicals. We used a laboratory toxicity experiment to assess variation in sensitivity to two pesticides among wood frog (*Lithobates sylvaticus*) populations and a mesocosm experiment to ascertain whether resistance to pesticides is associated with decreased performance when animals experience competition and fear of predation. We discovered that wood frog populations closer to agriculture were more resistant to a common insecticide (chlorpyrifos), but not to a common herbicide (Roundup). We also found no evidence that this resistance carried a performance cost when facing competition and the fear of predation. To our knowledge, this is the first study demonstrating that organophosphate insecticide (the most commonly applied class of insecticides in the world) resistance increases with agricultural land use in an amphibian, which is consistent with an evolutionary response to agrochemicals.

## Introduction

Global change often poses a major challenge for organisms because they must either move to regions that have more favorable environments or adapt to the novel conditions (Palumbi 2001; Meyers and Bull 2002). The use of agrochemicals, including pesticides, is one type of global change to which an increasing number of species are exposed as more land is being used for intensive agriculture (LeNoir et al. 1999; Hayes et al. 2010). Although we have made much progress in addressing the ecological consequences of pesticide exposure (Relyea and Hoverman 2006), the evolutionary consequences are poorly understood. The vast majority of evolutionary investigations are restricted to studies of target species, such as mosquitoes and crop pests, because evolved resistance poses economic and health concerns (Mallet 1989; Rosenheim et al. 1996). These studies have demonstrated that invertebrate pest species often evolve resistance, but pesticide resistance sometimes carries a fitness cost that may reduce the health of populations even after exposure to pesticides has ceased (Carrière et al. 1994; Coustau and Chevillon 2000). However, fitness costs are not always detected (Arnaud and Haubruge 2002; Bielza et al. 2008; Lopes et al. 2008), and studies reporting no costs may be false negatives because costs of resistance may only emerge when the stress of the pesticides is combined with natural stressors (Coors and De Meester 2008; Hardstone et al. 2009).

There is a growing awareness that nontarget species often experience collateral damage from pesticides, often resulting in death or sublethal effects on behavior, physiology, or endocrinology (Weis et al. 2001; Hayes et al. 2010; Jansen et al. 2011a; Tuomainen and Candolin 2011). In nontarget species, however, we know very little about evolved resistance (Jansen et al. 2011b). Moreover, studies on vertebrate species are rare (but see Boyd et al. 1963; Vinson et al. 1963), and we have no information for some of the most commonly applied pesticides (e.g. organophosphate insecticides).

Of the many taxonomic groups that are affected by global change, amphibians are a group that is experiencing global declines, with 32% of species threatened and 43% of species experiencing declines (Stuart et al. 2004). The causes of these declines are diverse, including habitat loss, disease, and introduced species. In some locations, these declines appear to be related to pesticide exposure and it is becoming increasingly clear that these stressors are more lethal when combined (Wake 1991; LeNoir et al. 1999; Stuart et al. 2004; Hayes et al. 2010). Only a few studies have addressed the impacts of pesticides on amphibians from an evolutionary perspective. Recently, a phylogenetic signal of pesticide sensitivity in amphibians was found for the organochlorine pesticide endosulfan (Hammond et al. 2012). While this study demonstrates that characteristics common to amphibian families (e.g. conserved physiology within clades) can predict sensitivity to endosulfan, studies at the individual and population levels are necessary to understand contemporary responses to pesticides. Population‐level studies have provided valuable insights on the tolerance of amphibians to nonpesticide toxicants (Persson et al. 2007; Brady 2012; Hopkins et al. 2012). Existing work on pesticides, restricted to the insecticide carbaryl, shows that amphibian species, populations, and individuals can vary in pesticide resistance and this resistance can carry a fitness cost (Bridges and Semlitsch 2000; Semlitsch et al. 2000; Bridges et al. 2001). However, no connection has been made between variation in resistance and patterns of land use. Only one study, using the insecticide DDT, has compared the resistance of amphibian populations from treated and untreated reference sites (Boyd et al. 1963). While the population from the pristine site was very sensitive, there was no clear mortality pattern for sites that were sprayed directly versus sites that probably experienced indirect exposure (e.g. drift or runoff). In addition, we have no information on whether amphibians can evolve resistance to major groups of pesticides that are commonly used today (e.g. organophosphate insecticides) or whether resistance varies across pesticides that have different modes of action.

We assessed whether wood frog populations vary in their resistance to the most commonly used insecticide (chlorpyrifos) and herbicide [Roundup Original MAX^®^ (active ingredient: glyphosate)] in the agricultural sector (Grube et al. 2011), whether the variation in resistance is associated with variation in agricultural land use, and whether sensitivity to pesticides is associated with adaptive responses to competition and the threat of predation. We collected newly oviposited eggs from nine populations across a land‐use gradient and reared them under common‐garden conditions prior to using tadpoles in experiments. First, using a standard toxicology experiment, we tested the hypothesis that populations of wood frogs collected from ponds in areas with more agriculture were more tolerant to moderately lethal concentrations of chlorpyrifos and Roundup. Second, using an outdoor mesocosm study, we tested the hypothesis that more resistant populations of wood frogs would have reduced performance (measured as fitness components including survival, larval growth rate, and size at metamorphosis and time to metamorphosis) and that such costs would be more pronounced under stressful conditions (i.e. the presence of predators or high competition).

## Methods

### Animal collection and husbandry

**Figure 1:**
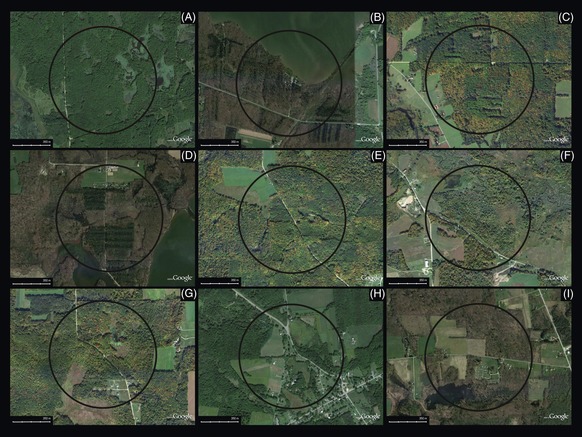
Arial maps showing surrounding land use for ponds. (A) Turkey Track, (B) Relyea, (C) Square, (D) Blackjack, (E) Road, (F) Log, (G) Bowl, (H) Graveyard, (I) Mallard. The circle represents a 500‐m radius around each pond.

Experiments were conducted at the University of Pittsburgh's Pymatuning Laboratory of Ecology during 2009 and 2010. Each year, we collected 9–10 recently‐laid egg masses (composed of early‐stage embryos) from each of nine wood frog populations. Wood frogs typically remain within 300 m of their natal pond and their genetic neighborhood is generally within 1 km of the breeding pond (Berven and Grudzien; Semlitsch 1998, 2000). In our study, the shortest distance between ponds was 4 km, so it is unlikely that animals from different ponds were from the same population. Ponds were chosen so that they varied in the amount of land nearby dedicated to the production of pasture/hay, row crops, and small grains (Fig. 1; see Figures S1 and S2 for a regional view of the ponds). For each pond, we considered both the distance to the nearest agricultural field and the proportion of land used for agriculture within a 500‐m radius of the pond. Egg masses were hatched in covered 200‐L plastic wading pools (4–5 egg masses per pool) filled with aged, untreated well water. Tadpoles were fed rabbit chow *ad libitum* until used in experiments.

### Assessment of pesticide resistance

In the spring of 2010, we conducted a 48‐h laboratory toxicity experiment using a completely randomized design to assess each population's sensitivity to chlorpyrifos and Roundup. We used published LC50 values and pilot studies to select nominal concentrations for each pesticide that would be moderately lethal to tadpoles (Sparling and Fellers 2007, 2009; Jones et al. 2009). Wood frogs from each of the nine populations were exposed to three treatments: (i) no‐pesticide control, (ii) chlorpyrifos, and (iii) Roundup (active ingredient is glyphosate). For chlorpyrifos, we used a nominal concentration of 1.75 ppm. For glyphosate, we used a nominal concentration of 2.5 ppm for the first 24 h. This concentration did not cause much mortality over the first 24 h of the experiment so we increased the concentration to 2.75 ppm for the second 24 h. These are the most commonly used insecticide and herbicide in agriculture (Grube et al. 2011) and among the most commonly used pesticides in the study area (2002 estimated usage: chlorpyrifos = ≥0.5 kg/km^2^; glyphosate = 0.6–2.6 kg/km^2^; http://water.usgs.gov/nawqa/pnsp/). The 27 treatment combinations were replicated five times for a total of 135 experimental units.

Working pesticide solutions were prepared using technical grade chlorpyrifos (99.5% purity; Chem Service, West Chester, PA, USA) and Roundup Original MAX^®^ (a formulation commonly used in agriculture). The water was carbon‐filtered and UV‐irradiated. Because chlorpyrifos is moderately insoluble in water, we used an ethanol carrier. Previous work has shown that the concentration of ethanol used (approximately 0.04% ethanol) does not affect tadpole survival; therefore, we did not include ethanol in the no‐pesticide controls or the Roundup solutions (Jones et al. 2009). Solution samples were sent to the Mississippi State Chemical Laboratory to ascertain actual concentrations. For chlorpyrifos, actual concentrations were 0.438 and 0.584 ppm for the day 1 dose and day 2 dose, respectively (detection limit = 0.01 ppm). For Roundup (i.e. glyphosate), actual concentrations were 3.218 and 3.675 ppm acid equivalents (a.e.) for the day 1 dose and day 2 dose, respectively (detection limit = 0.0075 ppm a.e.).

All embryos used in the experiment hatched within a 16‐h period after the last population was collected from the wild. When we initiated the experiment, tadpoles had a mass (mean ± SD) of 51 ± 6 mg (range among populations = 40–60 mg) and were at or near Gosner stage 25 (Gosner 1960). Groups of 10 tadpoles were randomly assigned to 70‐mL glass Petri dishes containing pesticide solutions. Individual tadpoles were not exposed to more than one pesticide treatment. Dishes were checked every 4 h and dead tadpoles were removed. After 24 h, the water was changed in all experimental units and the pesticides were reapplied. The experiment was terminated after 48 h. We used an analysis of variance (anova) to examine the effects of pesticide, population, and their interaction on 48‐h survival. Tukey's HSD test was used for multiple comparisons. Although population is usually used as a random factor in analyses of ecological data, we chose to use it as a fixed factor in our analyses. We were interested in whether the specific, representative populations that we chose based on surrounding land use differed in their mortality when exposed to pesticides. This required the inclusion of an interaction term to test the null that all populations had similar tolerances to pesticides, which required that both population and pesticide treatment be included as fixed effects in the model (Bennington and Thayne 1994; Hopkins et al. 2012).

### Assessment of population responses to predator cues and competition

To assess whether pesticide resistance had costs, we raised the nine populations from hatchlings to metamorphosis in outdoor mesocosms under three different environmental conditions. We used a completely randomized design that crossed the nine populations with three environments: control, predator cues, and competition. We applied the predator cue and competition treatments to assess whether performance costs were only evident under stressful conditions. The 27 treatment combinations were replicated four times for a total of 108 experimental units. The experimental units were outdoor mesocosms constructed of wading pools filled with 90 L of well water, 100 g of dried oak leaves (*Quercus* spp.), and 1 g of rabbit chow to serve as structure and nutrients for periphyton growth. Water containing algae and zooplankton was collected from nearby ponds, predators were removed, and 500‐mL aliquots were added to each pool. The local ponds used for collecting zooplankton were not used to collect amphibians for the study. Each pool also received a predator cage made of slotted drain pipe (8 × 10 cm) that was covered on both ends with fiberglass screen. Pools were covered with 60% shade cloth lids to prevent colonization by organisms and prevent the escape of metamorphosing frogs.

Treatments were assigned on May 11, 2009. For each population, initial tadpole mass was based on 20 randomly selected individuals that were not allocated to the experiment. For mesocosms assigned the predator treatment, we added a single larval dragonfly (*Anax junius*) to the predator cage and fed each predator three times a week using approximately 300 mg of wood frog tadpoles from a mixture of the nine populations. Cages in predator‐free mesocosms were briefly lifted out of the water to equalize disturbance across mesocosms. To manipulate competition, we stocked 20 tadpoles in the control and predator cue treatments and 40 tadpoles in the competition treatment. Similar manipulations have been used to induce adaptive changes in tadpole traits (Van Buskirk and Relyea 1998; Relyea 2002).

On 4 June, we sampled 10 individuals from each pool to assess larval growth rates. Most tadpoles were at an intermediate developmental stage (mean Gosner stage ± SD = 33 ± 4; Gosner 1960; Werner and Anholt 1993; Jones et al. 2010). Mean individual mass was quantified for each pool, and all animals were returned to their pools. We calculated larval growth rate by subtracting the initial average mass of tadpoles from the average mass on 4 June and dividing by the number of days into the experiment. Growth rates were log‐transformed.

On 5 June, we quantified tadpole behavior. Screen lids were removed 30 min prior to the observations. For each pool, we counted the number of tadpoles observed and the number moving during a 60‐s period. Ten observations were recorded for each pool (five in the morning and five in the afternoon). From these data, we calculated the mean proportion of tadpoles observed and the mean proportion of tadpoles that were active in each pool.

Metamorphs were first observed on 10 June and we then checked for metamorphs daily. Metamorphs were removed from pools and kept in a plastic container with a small amount of water until full tail resorption (Gosner stage 46). Metamorphs were then euthanized in MS222 and preserved in 10% formalin. On 31 July, we concluded the experiment. Percent survival and mean metamorph mass were calculated for each pool.

We used a multivariate analysis of variance (manova) to assess the effects of environment (control, with predator cues, and with increased competition) and population on tadpole performance (growth rate, time to metamorphosis, mass at metamorphosis, survival, activity, and refuge use), followed by anovas and Tukey's HSD pairwise comparisons. Population was included as a fixed effect in the model because we were interested in whether the specific, representative populations chosen based on their surrounding land use differed in their responses to the environment treatments (Bennington and Thayne 1994).

### Assessment of pesticide resistance across an agricultural land‐use gradient and its associated costs

We used regressions to assess whether pesticide resistance is correlated with agricultural land use and whether resistance is correlated with the traits that differed among populations in the mesocosm experiment. We did this by calculating two measures of agricultural land: the proportion of land used within 500 m of each pond and the linear distance from a pond to the nearest agricultural field. To derive these measures, we used satellite images from Google Earth Pro and a National Land Cover Data (NLCD) map that uses satellite imagery with a 30‐m resolution (http://landcover.usgs.gov/show_data.php?code=PA&state=Pennsylvania) (Vogelmann et al. 2001).

We calculated the proportion of land used for agriculture by defining an area encompassed by a 500‐m radius from the center of each pond. We chose a 500‐m radius because amphibians typically move <300 m from their natal pond (Semlitsch 1998, 2000) and the genetic neighborhood for wood frogs is generally within approximately 1 km (Berven and Grudzien 1990). Therefore, a 500‐m radius is likely to cover the area that juvenile and adult animals would travel and experience pesticides. This provides a conservative measure of pesticide exposure because it does not consider exposures from drift or runoff. In addition, agricultural fields >500 m from small ponds do not have strong effects on aquatic systems (Declerck et al. 2006). We overlaid Google Earth images with a NLCD map of PA, USA and extracted land used for pasture/hay, row crops, and small grains using Photoshop CS5 Extended (Adobe Systems Inc., San Jose, CA, USA). We summed these land‐use types and calculated the proportion of land used for agriculture. To improve linearity, the proportion of land used for agriculture was arcsine‐transformed.

We measured the linear distance from the center of a pond to the closest agricultural field. We did not differentiate among different types of agriculture, because farmers in our area rotate crops from year to year. An important point is that the landscape around aquatic habitats may affect runoff, which could weaken distance from the point source and proportion of land used for agriculture as indicators of exposure to pesticides (Schriever and Liess 2007). However, the complex life cycle of amphibians means that they could come into contact with pesticides both in water, as larvae, and on land, as juveniles and adults. Thus, topographical and landscape features that reduce runoff into breeding ponds may not eliminate the risk of exposure. To improve linearity, the distance to agriculture was log_10_‐transformed.

Inherent in this regression approach is an inability to pinpoint causation in the observed relationship. Experimental evolutionary approaches (e.g. quantitative genetic breeding designs and experimental evolution) would provide much needed data on the evolutionary implications of pesticide exposure to amphibians (Jansen et al. 2011b). Such approaches are difficult to employ in amphibians (due to complex life cycles and relatively long generation times), but would be invaluable in understanding the evolutionary implications of pesticide exposure to amphibians.

## Results

### Assessment of pesticide resistance

**Figure 2:**
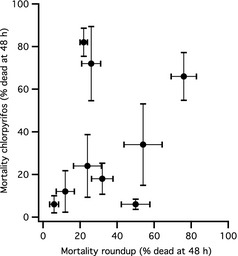
Variation among populations in sensitivity to pesticides. Results are 48‐h mortality estimates for the insecticide chlorpyrifos and the herbicide Roundup Original MAX ^®^. Data represent population means ± 1 SEM.

In the toxicology experiment, we found that survival in the controls was high (≥98%), and populations exposed to the pesticides varied widely in their sensitivity (Fig. 2). In addition, patterns of sensitivity were not consistent between chlorpyrifos and Roundup (population‐by‐pesticide interaction *F*_8,90_ = 5.031, *P *<* *0.001). There was tremendous population variation in sensitivity to chlorpyrifos (*F*_8,45_ = 6.17, *P *<* *0.001), with mortality ranging from 6% to 82%. Populations also varied widely in their sensitivity to Roundup (*F*_8,45_ = 10.637, *P *<* *0.001), with mortality ranging from 6% to 76%. There was no correlation between a population's sensitivity to chlorpyrifos and Roundup (Pearson's *r* = 0.398, *P *=* *0.289, *n* = 9). In addition, small differences in initial size had no effect on a population's sensitivity to either pesticide (both *P *≥* *0.239).

**Figure 3:**
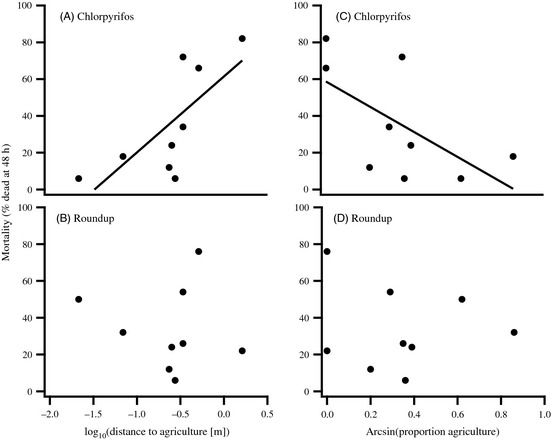
Relationship between the intensity of agricultural land use near ponds and a population's sensitivity to pesticides. Results are presented for (A, C) chlorpyrifos and (B, D) Roundup Original MAX ^®^. Markers represent population means. Proportion of land used for agriculture was calculated within a 500‐m radius of each pond. The fitted line for panel (A) was significant (*P *=* *0.026), whereas the fitted line for panel (C) was marginally nonsignificant (*P *=* *0.075).

In considering the agricultural context of each population, we found that populations located closer to agriculture were more resistant to chlorpyrifos than populations located far from agriculture (*P *=* *0.026, *R* ^2^ = 0.531; Fig. 3A). Additionally, populations with a higher proportion of land used for crops tended to be more resistant to chlorpyrifos than populations in more pristine areas (*P *=* *0.075, *R* ^2^ = 0.384; Fig. 3C). Interestingly, sensitivity to Roundup was not correlated with either land‐use variable (distance to agriculture: *P *=* *0.797, proportion of land used for agriculture: *P *=* *0.874; Fig. 3B,D).

### Assessment of population responses to predator cues and competition

In the mesocosm experiment, we found that tadpole behavior and life history traits differed among populations (*F*_48,438_ = 3.669, *P *<* *0.001) and among environments (*F*_12,138_ = 29.553, *P *<* *0.001). However, all populations responded to these environments in the same direction with similar magnitudes (i.e. there was not a population‐by‐environment interaction; *F*_96,438_ = 0.9, *P *=* *0.732). The population differences were large for many of these traits (Figure S3). Competition and predator cues had strong effects on tadpole behavior and life history traits (Table 1). Relative to controls and average across populations, predator cues increased tadpole refuge use by 12%, decreased activity by 12%, and increased time to metamorphosis by 7%. Competition increased activity by 10%, decreased larval growth rate by 14%, decreased mass at metamorphosis by 40%, and increased time to metamorphosis by 12% (Figure S4).

**Table 1:** anova results from the outdoor mesocosm experiment. Growth rate and behavior (activity and refuge use) were recorded midway in the experiment. Time to and size at metamorphosis and survival were recorded at the end of the experiment

Source	Growth rate	Time to metamorphosis	Mass at metamorphosis	Activity	Refuge use	Survival
Population df = 8, 90	**52.714** **<0.001**	**6.362** **<0.001**	1.763 0.098	0.86 0.554	**2.218** **0.036**	1.305 0.255
Environment df = 8, 90	**120.789** **<0.001**	**87.234** **<0.001**	**225.414** **<0.001**	**35.962** **<0.001**	**6.817** **0.002**	1.42 0.248
Population‐by‐environment df = 16, 90	1.351 0.191	0.886 0.587	0.504 0.937	0.903 0.569	0.423 0.972	0.882 0.591

Bold values are statistically significant.

**Figure 4:**
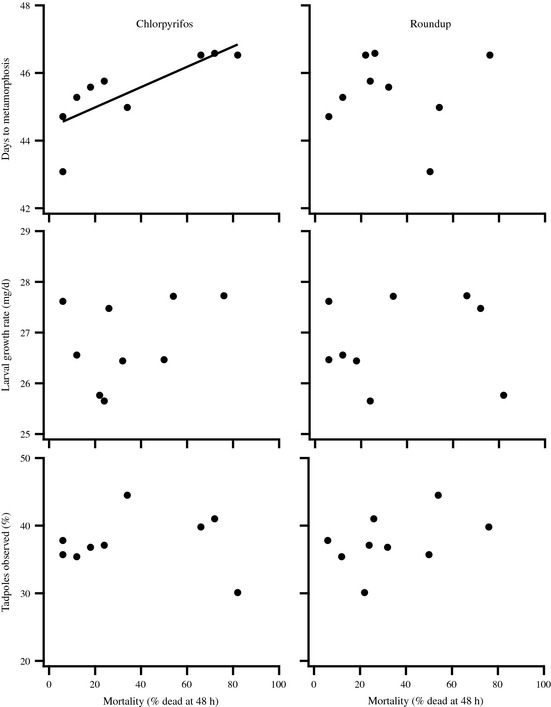
Relationship between a population's sensitivity to pesticides and life history and behavior. We present results for the three traits that differed among populations [days to metamorphosis, larval growth rate (mg/day), and proportion of tadpoles observed during behavior trials]. Data represent population means.

In the mesocosm experiment, we found no evidence that increased resistance was associated with fitness costs regardless of the environmental context. For Roundup, we found no relationship between a population's survival in the laboratory toxicity experiment and any of the wood frog traits expressed in the mesocosm experiment (all *P *≥* *0.244). For chlorpyrifos, we found no relationship between a population's survival in the laboratory toxicity experiment and either the tadpoles' growth rate or the number of tadpoles observed (growth rate: *P *=* *0.853; percentage of tadpoles observed: *P *=* *0.902). However, we did find that more resistant populations metamorphosed up to 4 day quicker (*P *=* *0.01, *R* ^2^ = 0.637; Fig. 4).

## Discussion

We found that wood frog resistance to chlorpyrifos and Roundup was quite variable across populations. For chlorpyrifos, this variation was associated with proximity of the population to agriculture; populations closer to agriculture had higher survival than populations farther from agriculture. A similar pattern, although not statistically significant, was found when we used the proportion of land containing agriculture within a 500‐m radius. In the mesocosm experiment, we found that predators induced lower activity and increased refuge use in wood frogs. This led to longer times to metamorphosis. We also found that higher competition, in the form of increased tadpole density, induced higher activity and decreased larval growth rate. This led to longer times to and smaller mass at metamorphosis. These results are consistent with previous studies that have addressed behavioral and life history responses to predator cues and increased competition (e.g. Relyea 2002). More importantly, there was population‐level variation in these traits. However, we found no evidence that populations with greater pesticide resistance paid a performance cost for resistance in pesticide‐free environments.

The chlorpyrifos results are consistent with an evolutionary response to insecticide exposure. Chlorpyrifos is the most commonly used insecticide in the agricultural sector with 3–4 million kg applied annually in the United States (Grube et al. 2011). Moreover, in our study area, chlorpyrifos is one of the most commonly used insecticides, and other insecticides that dominate the U.S. market (i.e. organophosphates) share the same mode of action as chlorpyrifos (Grube et al. 2011; http://water.usgs.gov/nawqa/pnsp/). The evolution of resistance could have occurred via selection imposed by chlorpyrifos or due to cross‐resistance (i.e. when resistance to one pesticide confers resistance to other pesticides as well) to many carbamate and organophosphate insecticides that share the same mode of action. Cross‐resistance to pesticides is commonly observed in pest species, and we have recently confirmed this to be the case in wood frogs (Georghiou 1972; Hua et al. 2013). Our results must be met with caution because we do not have pesticide concentration data for the ponds used in our study. It would be ideal to have long‐term chlorpyrifos exposure data for each pond, to confirm past exposures that selected for tolerance and the current exposure risk faced by wood frogs. Given that we did not have this information, agricultural land use is our best predictor of exposure to pesticides and is correlated with sensitivity to pesticides and reduced genetic variation in aquatic invertebrates (Coors et al. 2009).

We also found variation among populations in sensitivity to Roundup, but it was not correlated with agricultural land use. There are several potential explanations. First, a variety of herbicides are used in our study area (see pesticide usage maps: http://water.usgs.gov/nawqa/pnsp/usage/maps/compound_listing.php?year=02) and, unlike insecticides that often share a single mode of action, these herbicides have diverse modes of action. Thus, any evolution of resistance to other herbicides is unlikely to confer cross‐resistance to Roundup (Georghiou 1972). As a result, the relationship between agricultural land use and exposure to Roundup may be weak. Second, Roundup is the second most commonly used herbicide in the home and garden sector and the industry, commercial, and government sector and is commonly sprayed to control the growth of noxious plants (Giesy et al. 2000; Grube et al. 2011). Thus, populations far from agricultural land may still be exposed to Roundup due to nonagricultural exposures. Organophosphate pesticides are also commonly used in nonagricultural sectors; however, the agricultural sector by far uses the most herbicides and insecticides (herbicides: 83% of total use is agricultural; insecticides: 70%; Grube et al. 2011). Finally, we exposed tadpoles for a short period, at relatively high concentrations, and in an artificial environment, which may underestimate or overestimate each population's tolerance to pesticides (Relyea and Mills 2001; Suchail et al. 2001; Weis et al. 2001). Field experiments over relatively long periods would be valuable in confirming whether patterns of tolerance found in the current study hold when tadpoles are exposed to concentrations of pesticides found in natural ponds (Persson et al. 2007; Brady 2012).

We found no evidence that resistance to pesticides reduces an individual's performance in pesticide‐free environments. We found that populations that were more resistant to chlorpyrifos metamorphosed faster than populations that were more sensitive to the pesticide; however, this effect was small and did not result in a smaller size at metamorphosis (which is much more important to fitness; Smith 1987). It is likely that the challenges (competitors and fear of predators) experienced by tadpoles in our mesocosm experiment were more benign than those in nature where tadpoles must contend with a suite of competitors and the combined stress of competition and predation. Therefore, it is possible that under more adverse conditions a performance cost to tolerance may have been uncovered (Coors and De Meester 2008; Hardstone et al. 2009). However, the presence of predator cues and more competitors was sufficient enough to cause changes in life history traits and behavior that are indicative of predator‐induced and competitor‐induced stress. Our results suggest that there never was a cost of resistance to chlorpyrifos or that costs were originally present, but populations evolved secondary mechanisms that reduce the costs. Reduced fitness costs of pesticide resistance can stem from compensatory mutations including allele replacement or the evolution of modifiers that reduce fitness costs (Guillemaud et al. 1998). Alternatively, fitness tradeoffs associated with local adaptation (e.g. to pesticide exposure) may not be as common as once thought (Hereford 2009). Such costs are expected to occur due to rampant pleiotropy (Fisher 1958). However, empirical estimates of pleiotropy are often weak and it is becoming increasingly clear that phenotypic evolution is often modular in nature, which would result in lower costs for expressing alleles that confer pesticide resistance (Wagner and Zhang 2011).

Here, we show that while pesticides can have a number of harmful effects on nontarget species, amphibian populations may be able to evolve resistance to pesticides without costs to how they respond to competitors and predators. However, researches on the genetic mechanisms that confer resistance to pesticides are necessary because it is possible that maternal effects and epigenetic mechanisms contributed to variation among wood frog populations in tolerance to pesticides (Morgan et al. 2007; Klerks et al. 2011). Interestingly, even if this is the case, maternal effects can themselves be adaptive and epigenetic inheritance can provide future generations the capacity to tolerate pesticide exposures (Mousseau and Fox 1998; Vandegehuchte and Janssen 2011). Our results appear to be the first example showing that amphibian populations near agriculture are more tolerant to pesticides, a result that is consistent with the evolution of pesticide resistance.

## Data archiving statement

Data deposited in the Dryad repository: doi:10.5061/dryad.sj5b6.

## Supporting information


**Figure S1.** Arial map showing the location of ponds used in this study in the state of Pennsylvania, USA.

**Figure S2.** Arial map showing the location of ponds used in this study within NW Pennsylvania, USA. Ponds are found in Crawford, Erie, and Warren counties.

**Figure S3.** Variation among populations in life history and behavioural response variables averaged across predator cue and competition treatments. Data represent population means ± 1 SEM.

**Figure S4.** The effects of perceived predation risk and competition on life history and behaviour averaged across populations. Data represent treatment means ± 1 SEM.
