# Supplementary figures and images for: Proximity to agriculture is correlated with pesticide tolerance: evidence for the evolution of amphibian resistance to modern pesticides
Source: Evol Appl. 2013 Apr 30;6(5):832–41. doi: 10.1111/eva.12069 (PMC5779125; doi:10.1111/eva.12069)

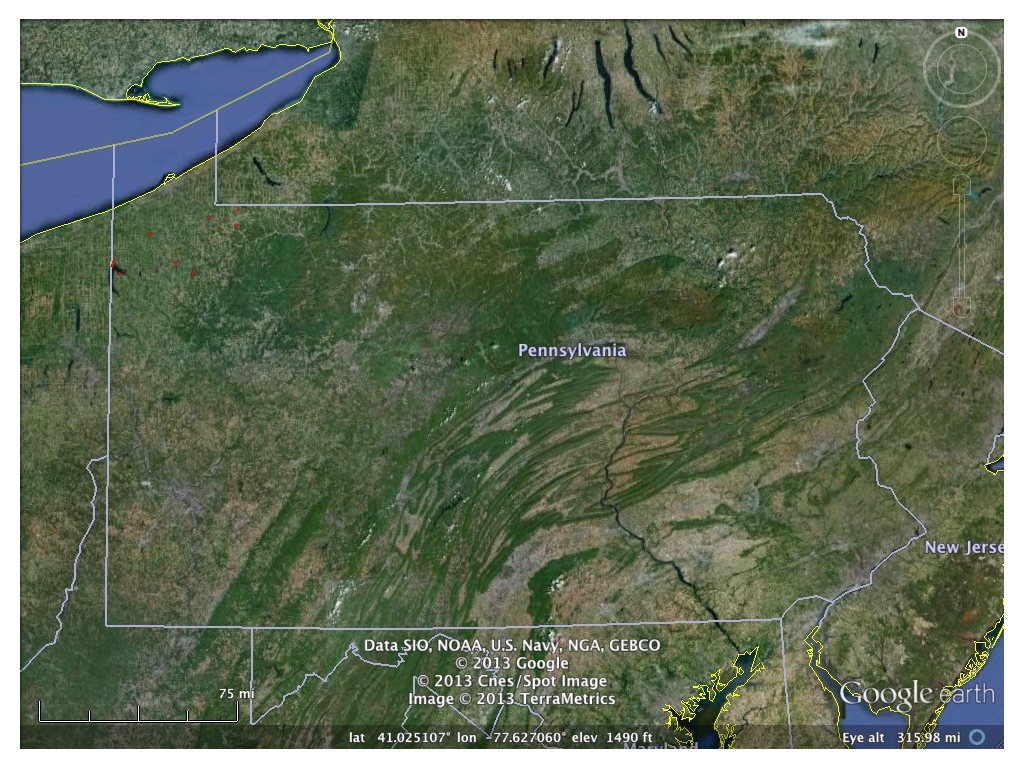

Supplement: Supplementary file 1 — Figure S1. Arial map showing the location of ponds used in this study in the state of Pennsylvania, USA. [file EVA-6-832-s003.tiff]

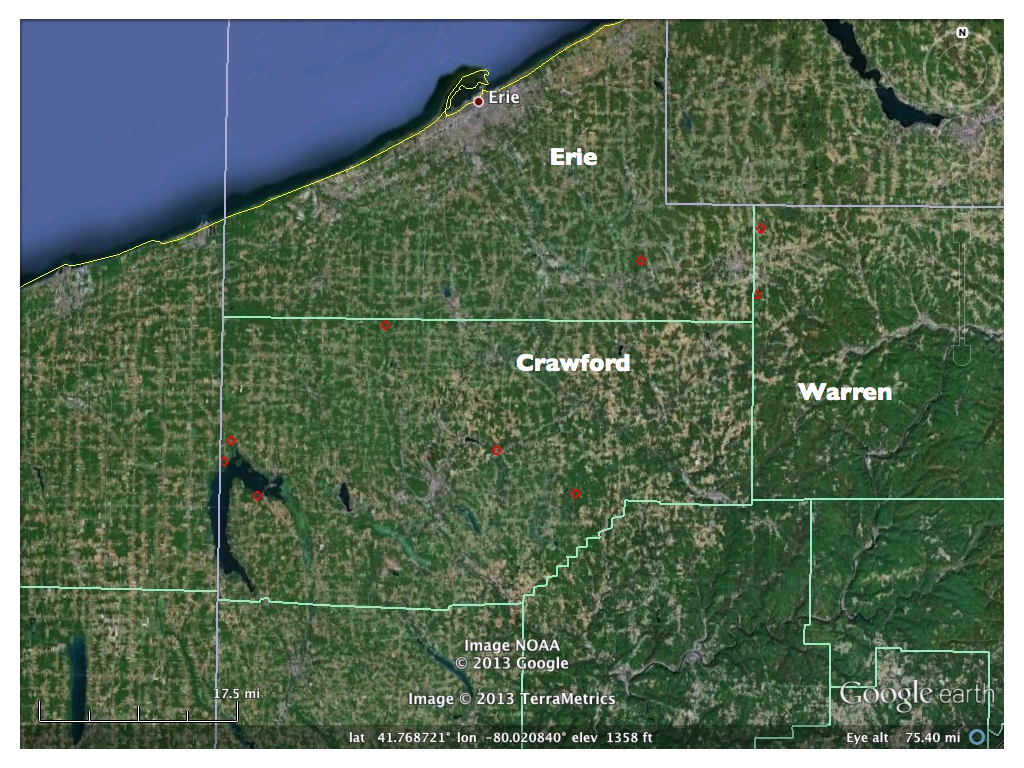

Supplement: Supplementary file 2 — Figure S2. Arial map showing the location of ponds used in this study within NW Pennsylvania, USA. Ponds are found in Crawford, Erie, and Warren counties. [file EVA-6-832-s002.tiff]

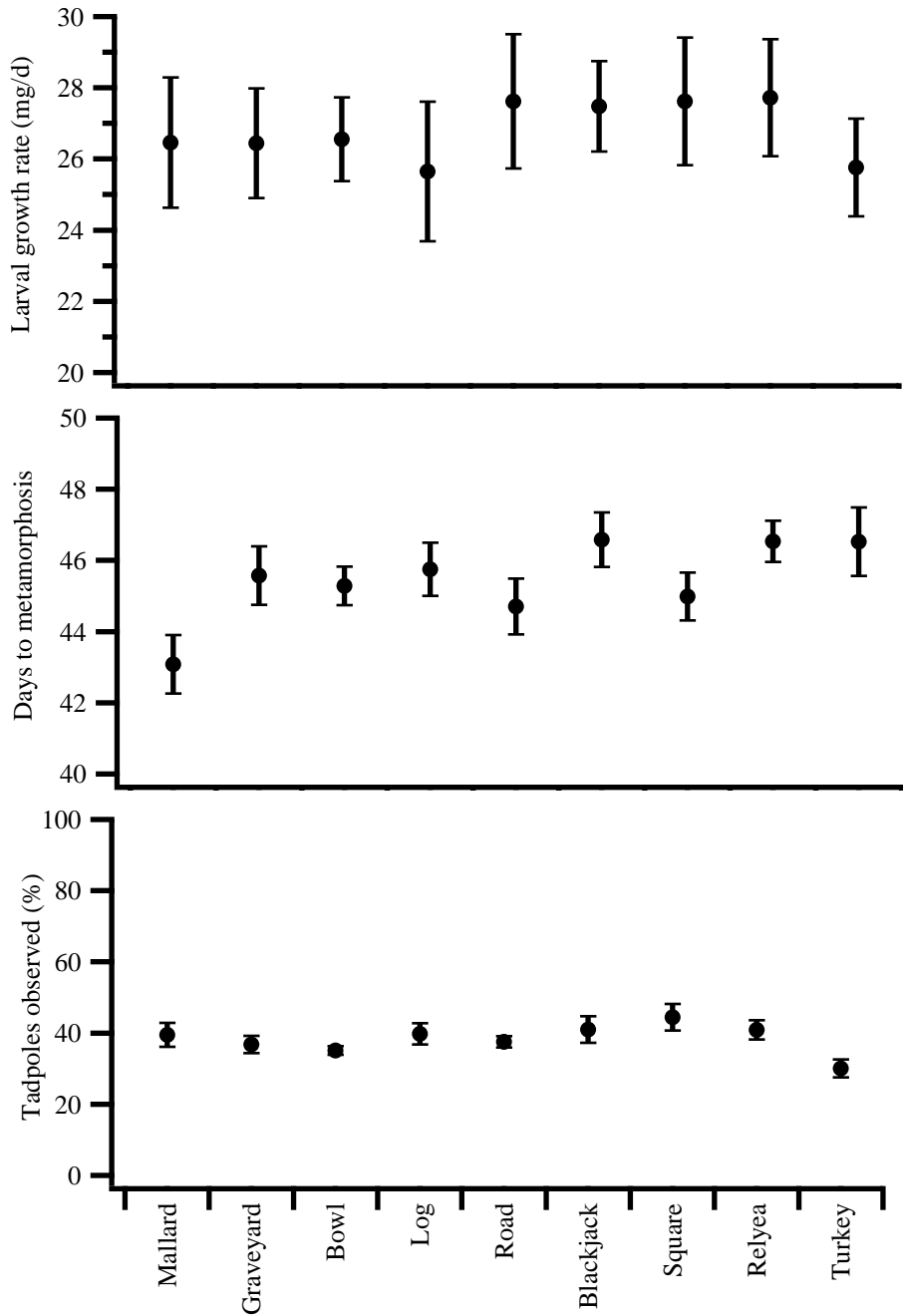

Supplement: Supplementary file 3 — Figure S3. Variation among populations in life history and behavioural response variables averaged across predator cue and competition treatments. Data represent population means ± 1 SEM. [file EVA-6-832-s001.pdf]

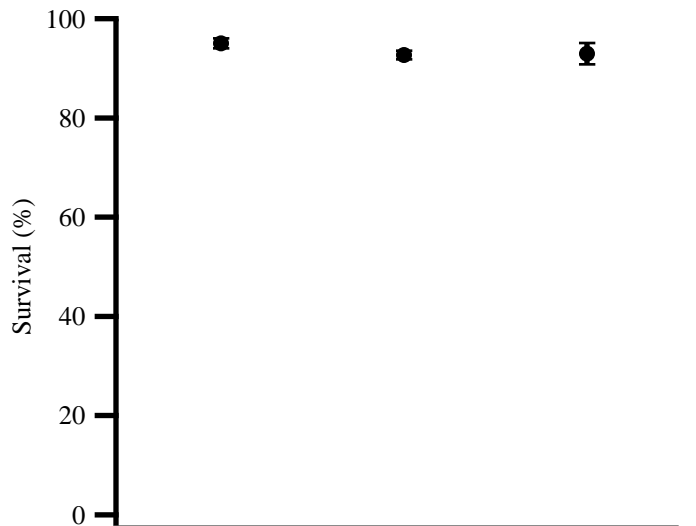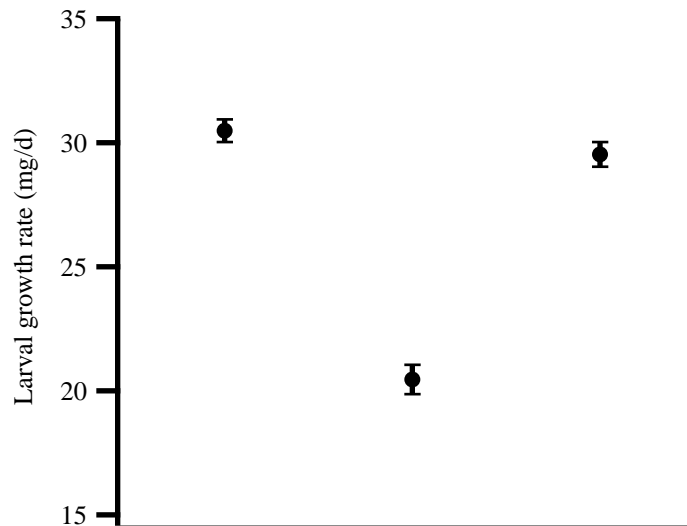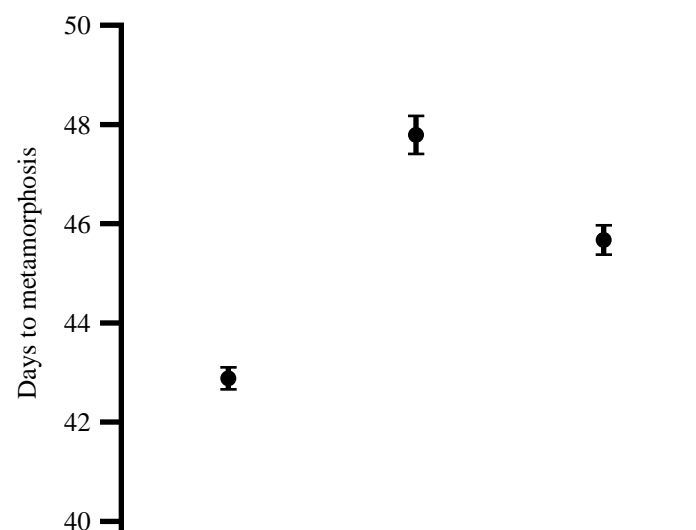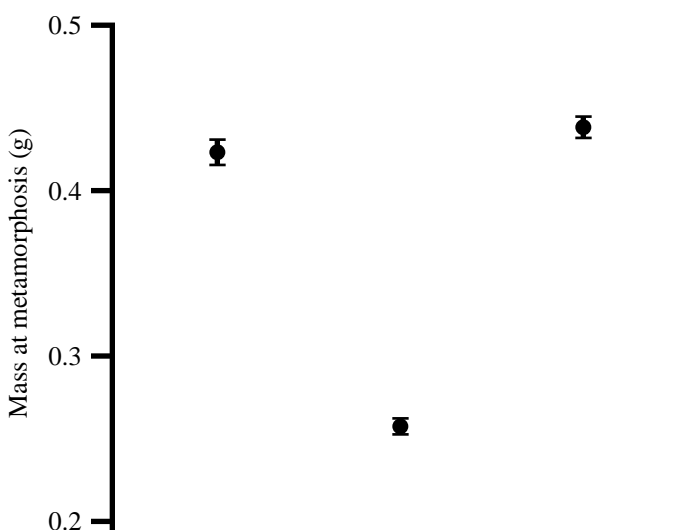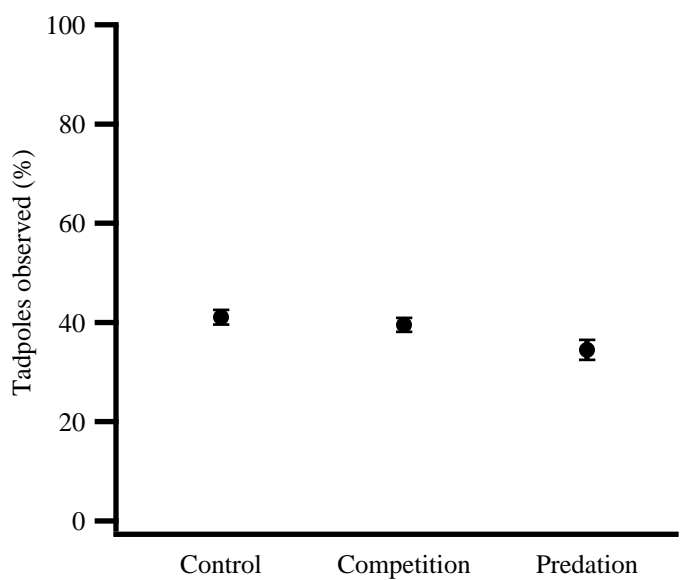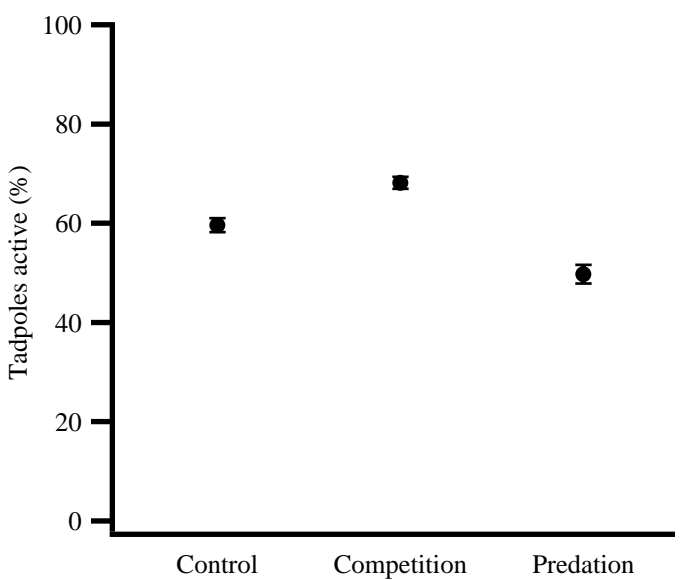

Supplement: Supplementary file 4 — Figure S4. The effects of perceived predation risk and competition on life history and behaviour averaged across populations. Data represent treatment means ± 1 SEM. [file EVA-6-832-s004.pdf]
