# Supplementary material for: Proximity to agriculture is correlated with pesticide tolerance: evidence for the evolution of amphibian resistance to modern pesticides
Source: Evol Appl. 2013 Apr 30;6(5):832–41. doi: 10.1111/eva.12069 (PMC5779125; doi:10.1111/eva.12069)
Supplement: Supplementary file 5 [file EVA-6-832-s005.doc]

**Figure S1. Arial map showing the location of ponds used in this study in the state of Pennsylvania.**

**Figure S2. Arial map showing the location of ponds used in this study within NW Pennsylvania.** Ponds are found in Crawford, Erie, and Warren counties.

**Figure S2. Variation among populations in life history and behavioural response variables averaged across predator cue and competition treatments.** Data represent population means ± 1 SEM.

**Figure S3. The effects of perceived predation risk and competition on life history and behaviour averaged across populations.** Data represent treatment means ± 1 SEM.
